# First reports of illegal fentanyl sales in Peru: the beginning of a crisis?

**DOI:** 10.3389/fpsyt.2025.1616610

**Published:** 2025-06-20

**Authors:** Aldo Alvarez-Risco, Shyla Del-Aguila-Arcentales, Neal M. Davies, Sandra Huamán-Pastorelli, Jaime A. Yáñez

**Affiliations:** ^1^ Facultad de Administración y Negocios, Universidad Tecnológica del Perú, Lima, Peru; ^2^ Sustainability and Business Research Group, Escuela de Posgrado, Universidad San Ignacio de Loyola, Lima, Peru; ^3^ Faculty of Pharmacy and Pharmaceutical Sciences, University of Alberta, Edmonton, AB, Canada; ^4^ Facultad de Ciencias Económicas, Universidad Nacional del Callao, Callao, Peru; ^5^ Escuela de Posgrado, Universidad Internacional Iberoamericana, Campeche, Mexico

**Keywords:** fentanyl, opioids, drugs, addiction, Peru, illegal

## Introduction

The 2021 report Opioids and New Psychoactive Substances in Peru, using a quantitative and qualitative analysis, revealed that 70.5% of respondents found it easy or very easy to access substances for abuse in Peru. The recent emergence of fentanyl has raised significant concerns among health and public safety officials. Fentanyl, a powerful synthetic opioid responsible for severe addiction and a high mortality rate in the United States, now poses a similar threat in Peru. Being up to 100 times more potent than morphine, fentanyl has become a leading cause of overdose deaths in North America, and its presence in Peru could signal the start of a comparable crisis.

## Control and selling situation of fentanyl

### USA context of fentanyl

North America has faced successive waves of opioid crises: prescription opioids in the early 2000s, heroin in the 2010s, and synthetic opioids—particularly fentanyl—since 2013. In 2022, fentanyl caused 200 deaths per day, bringing the total number of overdose deaths in the U.S. to more than 250,000 since 2018 (1); also, over 115 million pills containing illicit fentanyl were seized by law enforcement in 2023 (2). Meanwhile, the United Nations Office on Drugs and Crime (UNODC) has raised alarms about the proliferation of new psychoactive substances (NPS) globally, noting that Latin America has become both a transit region and an emerging consumer market for synthetic opioids (3).

### The Peruvian situation of fentanyl selling

Peru’s long-standing fight against narcotics has focused primarily on coca cultivation and cocaine production, with an institutional focus on illicit drug trafficking routes to North America and Europe. However, domestic consumption of opioids and other psychoactive substances remained relatively low until the COVID-19 pandemic. Peru, which suffered the world’s highest COVID-19 death rate, with 5,551 deaths per million, saw an increased use of fentanyl during the pandemic as part of the recommended sedation-analgesia protocols for patients on mechanical ventilation. This surge in fentanyl availability may have contributed to the growing risk of abuse. This medical demand, coupled with pre-existing regulatory gaps, created conditions conducive to diversion into illicit channels. Investigative reports and law-enforcement seizures indicate that an unknown proportion of leftover hospital fentanyl found its way into unregulated markets. In March 2025, Peruvian authorities confiscated 5,000 vials of fentanyl purportedly destined for the United States, seized in raids across the northern departments of Piura and Tumbes (4).

### Key drivers of illicit distribution

Despite official decrees requiring strict opioid control, many pharmacy owners and regional health‐authority offices lack the training and resources to verify prescriptions and track controlled‐substance inventories; also, laboratory analyses suggest that some fentanyl batches circulating in street markets derive from diverted medical‐grade product, while others are clandestinely manufactured in unregulated labs using precursor chemicals sourced via dark‐web channel (5). Surveys conducted in Lima and other urban centers show increasing patient and non‐medical demand for stronger analgesics and sedatives, partly driven by self‐medication to cope with chronic pain and mental‐health issues exacerbated by pandemic‐related stressors (6). Informal networks of street dealers have begun offering fentanyl‐laced heroin and counterfeit prescription pills, appealing to cost‐sensitive users seeking more powerful effects (7). Cross‐border smuggling through porous Andean corridors remains difficult to police comprehensively, creating vulnerabilities exploited by transnational criminal organizations.

## Discussion and recommendations

Preventive strategies against the illegal sale of fentanyl are outlined in the Multilateral Evaluation Mechanism: Evaluation Report on Drug Policies (8). The report emphasizes the importance of institutional strengthening, with Priority Action 1.3 recommending that the national drug authorities receive adequate resources to control fentanyl distribution. This is critical, as fentanyl is currently easily accessible in pharmacies, highlighting the need for local and national authorities to have sufficient human resources for effective enforcement. Priority Action 2.2 calls for stakeholder collaboration to update national drug policies based on scientific evidence. Surprisingly, the report notes that scientific experts are not sufficiently involved in shaping national strategies and regulations.

The response to the Multilateral Evaluation Mechanism: Evaluation Report on Drug Policies is active because Peru is focusing on institutional strengthening to address the emerging threat of fentanyl through actions such as those led by the National Commission for Development and Life without Drugs (DEVIDA), which collaborates with international organizations such as the United Nations Office on Drugs and Crime (UNODC), the Drug Enforcement Administration (DEA), and the Anti-Narcotics and Law Enforcement Affairs Section (INL) of the United States government (9); likewise, Peru’s drug regulatory agency called on fentanyl manufacturers and importers in 2025 to prevent its misuse, proposing regulatory changes that include reducing the packaging volume of bottles to facilitate control and traceability (10). Peru has intensified its participation in regional and international cooperation mechanisms, such as the Inter-American Drug Abuse Control Commission (CICAD), in order to share good practices, obtain technical assistance and coordinate multilateral responses to this threat (11).

Law 32250 from January 2025 was released to modify the penal code to strengthen the fight against illicit drug trafficking (12). Exactly, the law adds two specific aspects to fentanyl: aggravated forms, which indicate “a prison sentence of not less than fifteen nor more than twenty-five years, from one hundred eighty to three hundred sixty-five days-fine and disqualification when the drug to be marketed or marketed exceeds…….three milligrams of fentanyl or its analogues” and attenuated forms of production, marketing and possession, which indicate “a prison sentence of not less than three nor more than seven years and from one hundred eighty to three hundred sixty days-fine when the quantity of toxic drug produced, manufactured, extracted, prepared, marketed, delivered to third parties or possessed for illegal uses by the agent does not exceed…….up to one milligram of fentanyl.” Initially, the law only mentioned cocaine base paste or its illicit derivatives, cocaine hydrochloride, opium latex or its derivatives, marijuana or its derivatives, Methylenedioxyamphetamine – MDA, Methylenedioxymethamphetamine – MDMA, Methamphetamine or similar substances.

Also, in March 2025, a mega-operation seized the largest amount of fentanyl in Peru: from state pharmacies to the black market for $600,000. The new regulation could be useful, but it needs to include more technological tools to be effective; in this way, AI algorithms can systematically scan social‐media posts, encrypted messaging‐app groups, and darknet forums to detect keywords, slang, and transactional patterns indicative of fentanyl sales: then, machine‐learning models could analyze national prescription‐dispensation databases to identify outliers—such as unusually high volumes of fentanyl per prescriber or region—and trigger audits or alerts. Additionally, customs and border‐control agencies can deploy AI‐powered imaging systems to inspect shipping parcels and cargo containers, using trained neural networks to recognize packaging anomalies or concealments commonly associated with drug shipments; finally, chatbots hosted on official health‐department websites, social‐media platforms, and popular messaging apps can disseminate interactive, multilingual information on the dangers of opioid use, overdose‐prevention strategies, and where to access treatment services.

### Urgent actions and proposals

Among the urgent actions being taken, the Ministry of Health’s meetings with representatives from various institutions specializing in health, addiction, and drug regulation are recognized to strengthen the prevention of inappropriate fentanyl use in the health sector (13). Previously, the regulatory and inter-institutional coordination efforts underway to control fentanyl abuse have been described; however, some urgent actions are needed to achieve a short-term impact. Thus, the authors propose implementing a national fentanyl prescription monitoring system to track atypical volumes in real time and send automatic alerts to authorities. Simultaneously, all pharmacists and prescribing physicians will have one year to certify their competencies in the safe handling of opioids and the detection of counterfeit products. Additionally, a quarterly meeting will be held between the drug regulatory agency, professional associations, health institutions, and the national police to understand the institutional consumption profile, the results of fentanyl seizure operations, review the proposed prevention standards, and the results achieved so that the joint effort can be strengthened based on results. It is recommended that, based on citizen science methodology, the public actively participate in reporting illegal fentanyl sales so that authorities can take the necessary control measures.
